# Infants’ Individuation of Faces by Gender

**DOI:** 10.3390/brainsci9070163

**Published:** 2019-07-11

**Authors:** Charisse B. Pickron, Erik W. Cheries

**Affiliations:** 1 Institute of Child Development, University of Minnesota 51 E River Pkwy, Minneapolis, MN 55455, USA; 2 Department of Psychological and Brain Sciences, University of Massachusetts Amherst 135 Hicks Way, Tobin Hall Amherst, MA 01003, USA

**Keywords:** individuation, gender, infants, face processing, manual search task

## Abstract

By 3 months of age, infants can perceptually distinguish faces based upon differences in gender. However, it is still unknown when infants begin using these perceptual differences to represent faces in a conceptual, kind-based manner. The current study examined this issue by using a violation-of-expectation manual search individuation paradigm to assess 12- and 24-month-old infants’ kind-based representations of faces varying by gender. While infants of both ages successfully individuated human faces from non-face shapes in a control condition, only the 24-month-old infants’ reaching behaviors provided evidence of their individuating male from female faces. The current findings help specify when infants begin to represent male and female faces as being conceptually distinct and may serve as a starting point for socio-cognitive biases observed later in development.

## 1. Introduction

Investigations have extensively focused on when, how, and what information is perceived and processed from faces at an early age. From birth (and potentially prenatally, [1]), infants display a visual attention bias for faces and face-like configurations that supports early learning and allows for infants to readily perceive differences in face identities [2]. Our face perception capabilities sharply tune to the faces of individuals who share the gender and race of our primary caregivers [3,4]. Although much is known about infants’ abilities to perceptually distinguish faces, relatively few studies have examined how these perceptual capabilities develop into meaningful conceptual distinctions about people or groups that children eventually possess. The goal of the current study was to examine when infants begin to use conceptual distinctions to keep track of different types of people. For instance, infants may readily distinguish between the perceptual features of different genders’ faces, but do infants then conceptually represent these differing faces as belonging to distinct ‘kinds’ or categories of people? The current study addressed this open question by examining whether infants represent faces as conceptually distinct along two different dimensions: ontological kind (human vs. non-human) and gender (male vs. female). Bridging this gap between early perceptual capabilities and higher-level representations is a critical step in understanding the development of social categories and biases more generally.

### 1.1. Face Perception during Infancy

Infants’ earliest perceptual biases for faces seem largely driven by the sex of the infants’ primary caregiver, which is most often a female face [5,6]. This female face bias is even found in some nonhuman primate species [7]. Infants spend the majority of their time with adults who are of the same gender, race, and age as their primary caregivers [8,9]. Highly salient people in infants’ lives are experienced across different social contexts and locations, indicating that the quality and consistency of interactions may play important roles in developing both perceptual and conceptual representations of faces [10]. It is hypothesized that, through almost exclusive experience with caregivers, the rapid amount of social learning that infants experience in the first few years of life results in highly tuned capabilities [11,12,13,14].

Biases based on gendered facial features have been demonstrated using several perceptual tasks including spontaneous looking-time preferences, visual-paired comparisons tasks, and categorization tasks. The overall finding is that infants raised by females develop a visual system that robustly perceives and processes features in female faces to allow for efficient differentiation [15,16]. Neonates differentiate their mother’s face from similar looking female faces [6,17] and reliably discriminate among two similar looking female faces by 3 months of age [18]. Three- and 10-month-old infants look longer towards female than male faces [5,19,20,21] and do this even for prototypical girl versus boy face models [22]. Interestingly, infants raised primarily by males look longer at male faces than female faces [5]. When male and female faces are compared within different race groups, infants only display a female face bias for the race group that is most familiar [20,21,23,24]. These looking preferences indicate that infants’ perceptual biases for female faces generalize across age (children and adults) but are specific to a familiar race group. Older infants begin to display reliable differentiation capabilities for male faces. However, electrophysiological responses indicate infants maintain specialized neural sensitivity for female faces [25,26]. These biases for female faces are not permanent and can be shifted when the primary caregiver becomes a male even during the second half of the first year of life [27].

Representing multiple exemplars that are both distinctive and have shared common features reflects the process of categorization and is observed early in infancy [12,15,28,29,30]. Infants seem to first form a perceptual category for female faces [15]. This indicates the formation of a ‘female face’ prototype [31,32,33], which includes faces varying by race [33]. Female face categorization may also include other qualities that mark distinctions between males and females, such as face–voice matching [34,35,36,37,38], as well as body representations [39]. It is possible that these other qualities that are matched with ‘femaleness’ or female faces are precursors to conceptual representations of females being distinct kinds of people from males.

### 1.2. Beyond Perceptual Discrimination

The research described above provides clear evidence for infants developing and fine-tuning their facial discrimination capabilities from an early age. Infants appear to quickly construct perceptual categories based on the salience and similarity of features that appear consistent across stimuli. This capability is critical for supporting learning across development. However, one of the hallmarks of more mature categorization is the ability to group things based upon conceptual similarities, often in spite of there being superficial differences between members within the category. In other words, individuals that belong to the same ‘kind’ share properties that make them more conceptually similar, despite whatever superficial differences may exist [40,41]. When it comes to the perception of faces, it remains unclear how infants represent the variations found within and across gender. Do infants automatically represent faces they perceptually discriminate as belonging to distinct ‘kinds’ of people? Conversely, do infants possess conceptual categories that group together faces that differ perceptually from one another? One way of disentangling this distinction is by using paradigms that typically elicit a more conceptual construal in the infants’ mind. For example, paradigms used to examine the process of ‘individuation’, which is the ability to represent objects as distinct individuals that exist separately across space and time [41,42,43,44]. This process is a common topic of study across development, from young infants to adulthood [45,46,47]. One common theme across many individuation studies is how these judgments can be driven by the conceptual differences between objects, such as the type or ‘kind’ of entity something is. The logic or theory of this process is that two entities will be represented even more distinctly if they belong to different conceptual kind categories. For example, a seminal study by Xu and Carey (1996) [48] used a violation-of-expectation paradigm with 10- and 12-month-old infants using stimuli that represented different conceptual kinds of objects (e.g., duck, ball, truck). These stimuli were presented in spatiotemporally ambiguous events, such that only kind or property information could be used to individuate the number of objects involved. Twelve-month-old, but not 10-month-old, infants displayed longer looking towards the unexpected, single object outcome [48]. Critically, this result was not based upon the younger infants failing to notice the perceptual differences between the two objects (e.g., the duck and the truck). Instead, the fact that 10-month-olds did not individuate the two objects suggests that they might have ignored the perceptual differences between the objects and represented them in a more conceptual manner as belonging to the same category, ‘object’. Converging evidence for kind-based object individuation has been obtained using a manual search task [49,50,51,52]. Reaching duration as a dependent measure is hypothesized to index infants’ search for objects hidden from view, as this action challenges infants to act on information that they can recall [50]. Thus, this measure is proposed to be a more explicit index of the number of objects believed to be involved in the scene, indicating the process of individuation. In one version of a manual search task, infants either saw two objects appear on top of a box simultaneously (a spatiotemporal condition) or sequentially (a kind condition), and the reaching time inside of the box was measured as an index of infants’ representations of the number of objects involved in the event [50]. The results from this task mirrored prior studies using looking-time procedures [48]; 10-month-old infants individuated objects solely based on spatiotemporal information whereas 12-month-old infants displayed spatiotemporal and property–kind based object individuation.

### 1.3. Individuation of Natural Kinds

Infants’ possession of kind concepts for several rudimentary objects (e.g., a book, duck, truck, and ball) coincides with the time when they understand noun labels for such objects [43,48,52,53,54]. However, evidence indicates that infants possess concepts for even more basic kinds even without having word knowledge. For example, 10-month-old infants can individuate based on whether or not something belongs to the kind category, ‘animate agent’ [55]. Recent research indicates that infants as young as 6 months of age are found to have improved working memory capabilities when tested on tracking a doll’s face compared to a non-face object, suggesting that concept knowledge for human faces aided infants’ memories of the presented events [56]. Other studies have found that 10-month-old infants individuate objects based on the biological kind ‘human’ [57,58]. Using humanlike dolls, infants displayed individuation of these objects from animal dolls (e.g., a dog) but did not show evidence of individuating two human dolls with varying features, such as skin color, gender, or hair, suggesting that they viewed male and female looking dolls as the same general kind [57]. One important consideration for the null gender effects is that the stimuli used were only caricatures of human faces. The dolls varied in surface material (e.g., rubber and porcelain), skin color, length of hair, and clothes (e.g., the presence of a hat), making it difficult to determine if the null results were due to the stimulus not being realistic. Therefore, it is possible that infants’ sensitivities to gender could be more effectively revealed using realistic images of faces. Although perceptual biases for different types of faces are present as early as 3 months of age and persist into adulthood, it is unclear whether infants develop more refined conceptual properties for human faces based on gender. This question was addressed using individuation techniques with real human faces in the current study.

### 1.4. Current Study

Face gender is a highly salient perceptual marker in infants’ environments. For many infants, their first years of life are spent primarily with females, which is believed to be a critical driver for fast occurring changes in face gender processing capabilities and face categorization. The current study investigated if and when this perceptual cue is used to represent people as individual members of distinct kind categories. Early and extensive experience with female faces may also be a catalyst for forming conceptual representations about male and female individuals, yet the research reviewed thus far has not been able to clearly assess this possibility. The current study uses evidence that by 12 months infants’ face processing capabilities have tuned to faces based on face gender and have formed rudimentary conceptual knowledge of basic natural kinds (e.g., animacy and humanness) as a platform to investigate whether infants use facial features indicative of different gender groups to track distinct individuals in a scene. Specifically, we examined whether infants use facial features that vary across maleness and femaleness to distinguish one individual as conceptually distinct from another individual. The current study included two age groups (i.e., 12-month- and 24-month-old infants), as well as two conditions, to test the development of human face individuation using a manual search task [49,50,51,59,60]. The goal of Condition 1 was to determine if we could replicate infants’ individuation of human versus nonhuman stimuli using photographs of people. Condition 2 assessed whether infants have subordinate level representations of faces by presenting them with male versus female faces.

If infants interpret the perceptual differences between faces as reflecting different ‘kinds’ of individuals, it was predicted infants would expect two faces to exist in the event and show greater reaching to an unexpected outcome of one face versus two. Alternatively, if infants represent facial features of humanness or gender as malleable expressions of a single individual, then it was predicted that infants would not reach differently for one or two faces hidden in a box. Based on prior findings [57,58], it was predicted that both 12- and 24-month-old infants would individuate human from non-human faces. Evidence from both the face processing and social cognitive literature indicate that face gender is an early and consistent marker for infants and children to group individuals together [15,61]. Thus, we also predicted that infants would individuate male and female faces. Infants have perceptual categories for female faces by 3 months and male faces by 9 months. Based on these findings, combined with evidence that 12-month-old infants display kind-based individuation of objects, it seemed plausible that both 12- and 24-month-old infants in the current study would display individuation of male and female faces. However, it was also possible that older, but not younger, infants would show more robust individuation based on face gender, as by this age, infants have a larger vocabulary to describe people in their environment (e.g., mommy, daddy, boy, and girl).

## 2. Materials and Methods

All reported protocols were approved by an Institutional Review Board at the University of Massachusetts Amherst. Parents received a $15 cash payment for participating in this study.

### 2.1. Participants

#### 2.1.1. 12-Month-Olds

A total of 48 twelve-month-old infants participated in Conditions 1 and 2. Of these infants, 38 were included in the final sample. The remaining infants were not included due to fussiness and not completing the minimum number of 6 out of 8 trials (7), experimenter error (2), and/or computer error (1). The final sample of 11- to 12-month-old infants had an average age of 368.79 days (*SD* = 12.66) with 18 males and 20 females.

According to caregiver reports, 35 infants were racially identified as Caucasian and 3 were Multiracial. Ethnically, 35 of these infants were reported as not Hispanic or Latino, 2 were Hispanic or Latino, and 1 did not report. Infants came from families with an average education level of a four year degree, with an average income of 55–65 thousand dollars per year (*SD* = $25–$35 thousand).

#### 2.1.2. 24-Month-Olds

A total of 46 twenty-four-month-old infants participated in Conditions 1 and 2. Of these infants, 33 were include in the final sample. The remaining infants were excluded due to becoming fussy and/or not completing the minimum of 6 out of 8 trials (10), experimenter error (2), and/or computer error (1). The average age of the final sample was 693.48 days (*SD* = 71.76), with 17 males and 16 females.

Based on the reported demographic data, 28 infants were reported to be racially White or Caucasian, 2 were Middle Eastern, 1 was African American or Black, 1 was Asian, and 1 was Multiracial. The ethnic representation in the sample included 29 infants as not Hispanic or Latino, and 4 who were. Infants came from families with parents who had completed an average of some graduate-level education, with an average household income of 55–65 thousand dollars per year (*SD* = $15–$25 thousand).

### 2.2. Stimuli and Apparatus

**Figure 1:**
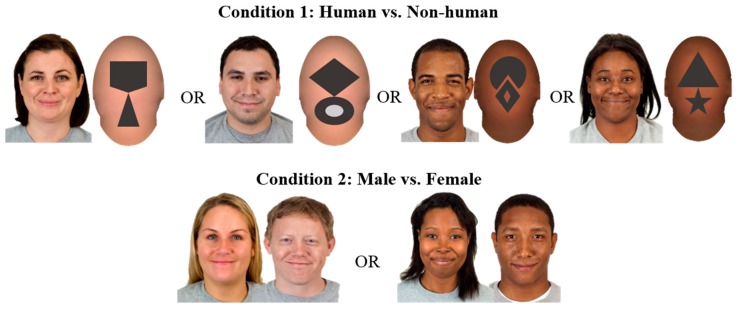
Example of face identities. Condition 1 infants were presented with human versus non-human images on foam-core blocks. Faces were either male or female paired with non-human image. Condition 2, infants were presented with male versus female faces. In both conditions infants either saw Caucasian or African American faces. There were a total of 6 different face identities for each face race group.

Images of human faces were chosen from the Chicago Face Database [62], with adults between 18- and 40-years-old. Faces were categorized based on race, gender, and rated for friendliness and attractiveness. All faces used in this study expressed a happy closed mouth smile. Half of the infants saw faces that were Caucasian, and the other half of infants saw African American faces. This stimulus difference was included with consideration of previous face perception findings that race can intersect with an infant’s processing of male and female faces [20,21,23,24]. A final set of 6 male and 6 female Caucasian and African American faces were used (see Figure 1). Faces were selected based on facial expression clarity, visibility of the eyes, and no apparent makeup, body piercings, or physical abnormalities. All male faces included in the final set had a hair length that did not extend past their ears, and most female faces had a hair length that reached to at least their chin. Three non-human-shaped faces were created in Photoshop CS5 12.0.x64 and Powerpoint (see Figure 1). The non-human-shaped stimuli were edited versions of the human faces. In Photoshop, the internal features of a Caucasian and an African American face were smoothed over and replaced with 2 non-human shapes (e.g., a triangle and a star). Three Caucasian non-human-shaped stimuli were used with infants who saw Caucasian faces and 3 African American non-human-shaped stimuli were used with infants who saw African American faces.

Faces measured 3 inches (tall) by 2 inches (wide) and were affixed to a block of stiff black foam core that was approximately 1 inch thick to allow for easily grasping of faces from within the box. Faces were affixed to both sides of the foam core block so that infants had plenty of access to the faces once they removed them from the box. The box was made of black foam core and was 10 (wide) × 5 (tall) × 12.6 (deep) inches. The front of the box had an opening that was 4 inches tall and 3 inches wide. The top and bottom half of this opening was covered by two pieces of red spandex sheet so that infants could not see into the box without having to reach in or separate the fabric. These box dimensions are consistent with previous object individuation manual search box tasks [50,59]. Two additional toys (i.e., a squeaky frog, squeaky duck, and koosh ball) were used during baseline trials. These objects were similar in dimension to the faces used during the manual search task.

### 2.3. Procedure

**Figure 2:**
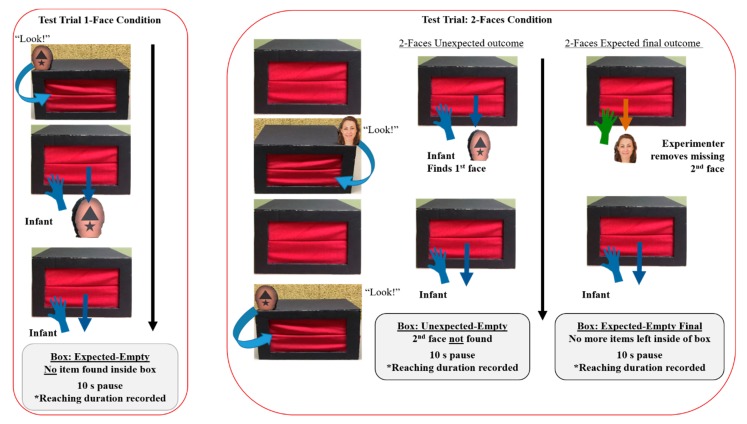
Manual search box task. Infants completed 8 trials, 4 trials per face block. There were three types of face outcomes. The image on the left depicts the expected-empty trial. Such that infants saw one face go in and only found one face within the box. The image on the right depicts the unexpected-empty and expected-empty-final trials. Unexpected-empty trials included infants seeing two faces go inside of the box but only finding one face. Expected-empty-final trials followed unexpected-empty trials. Infants were presented with the second face, leaving the box completely empty.

Parents were instructed to remain neutral and to not respond in any way to the events that occurred during the box task. Infants sat on their parent’s lap in front of a square table that was 33 inches wide and 45 inches tall. The individuation box task consisted of a baseline phase and a test phase (see Figure 2). All primary caregivers completed an in-depth demographics questionnaire. This questionnaire was used to gather quantitative data about infants’ experiences with males and females, as well as infants’ gendered word knowledge. For word knowledge, parents reported if they believed their infant comprehended: mom, dad, lady, man, woman, aunt, uncle, boy, girl, sister, brother, grandmother, and grandfather.

Baseline trials were used to familiarize infants with the action of removing items from within the box. Once the infant independently reached and retrieved a toy two times in a row, the experimenter continued to the test phase. During the test phase, the experimenter demonstrated the removal and replacement of faces from within the box. Eight test trials were divided into two blocks of 4 trials. One block of trials was used to evaluate infants’ individuation of the kind ‘human’ relative to non-human-shaped faces (i.e., Condition 1). The other block of trials was used to evaluate infants’ individuation of male versus female human faces (i.e., Condition 2). The test trials either involved a single face being removed and replaced or two faces being sequentially removed and replaced inside the box (see Figure 2). Each test trial contained a different set of face identities.

During the single face test trials, the experimenter pulled out one face object from within the box, placed the face on the far right corner of the front of the box, said “look,” and pointed at the face for approximately 5 s. The experimenter then picked up the face wiggled it in front of the infant and placed it back down on the left-front corner of the box once again saying “look,” while pointing. If needed, experimenters called the infants’ name after saying “look” to draw infants’ gaze towards the stimuli. Experimenters did not advance through the trial unless infants looked towards the face. After approximately 5 s, the experimenter replaced the face back inside the box, making sure the infant watched as the face went in the front. The box was then pushed towards the infant so they could retrieve the hidden face. Experimenters made sure the box was within an arm’s length for each infant, to maximize the accessibility of the box. If infants did not independently retrieve this first face, the experimenter would do so, showing the infant that it was possible to find something within the box. Reaching duration data from these “failed” trials were not included in the final analyses. These failed trials were not repeated. Instead, the experimenter completed the remaining steps of the trial. Regardless of whether or not the infant or the experimenter found the first hidden face, infants then had 10 s to observe or play with the face. Experimenters made explicit efforts (e.g., saying “look at this” or calling the infant by name) to redirect infants’ visual attention towards the stimuli during this 10 s period. The experimenter then took the face away while keeping the box within reach. Infants’ reaching behaviors were observed prior to and during a 10 s period after the experimenter took the face away. During this 10 s pause, the experimenter did not interact with the infant and kept their eyes down toward the table. This 10 s pause has been used in previous manual search box tasks [50,59].

For the two face stimuli test trials, the same actions were completed as in the single face trials. However, a second object (either a human face or a non-human face) was removed from the box once the first face stimuli were replaced. Infants never saw two faces come out of the box at the same time. Therefore, they did not receive any spatiotemporal cues for the number of items being hidden within the box. After the first face was shown and placed inside of the box, the experimenter indicated that their hand was empty and then retrieved a second face. This face was once again placed on top of the box in each corner and then replaced back inside of the box. The box was then pushed forward toward the infant. As this happened, the experimenter secretly removed one of the two faces out the back of the box so that the infant could only find one face. After the first face was found and infants had 10 s to examine the face, experimenters took the face away and allowed for a 10 s pause to measure reaching activities. Next, the experimenter reached inside the box and removed the second face, showing the infant that there was something leftover in the box. Once again, infants had 10 s to explore this second face. The face was then removed, followed by a final 10 s pause. The logic of having two types of test trials was to compare infants’ reaching behaviors when the box should have been completely empty (i.e., during a single face trial) to when the box was “unexpectedly” empty. If infants individuated the two different faces, then it was expected that they would display greater reaching when they could not find the second face (i.e., an unexpected-empty outcome) compared to when they only saw and found a single face (i.e., an expected-empty outcome).

Four components of the task were counterbalanced across 8 different versions. First, the block order was counterbalanced such that half of the infants completed the human versus non-human block (i.e., Condition 1) first, and the other half completed the male versus female block (i.e., Condition 2) first. Second, the trials within each block were presented in one of four types of patterns: 1, 2, 2, 1; 2, 1, 1, 2; 1, 2, 1, 2; or 2, 1, 2, 1. Infants never saw the same trial order across the two blocks. Third, the order of face pair presentation was randomized across the 8 counterbalance versions. Specifically, human versus nonhuman, as well as male versus female, face pairs were previously determined using a random pair generator, however the order of these face pairs was randomized across counterbalance versions. Fourth, the presentation order of face identity was randomized across 8 counterbalance conditions, such that infants did not see the same face identity in Conditions 1 and 2. Half of the infants saw Caucasian faces and the other half saw African American faces for each Condition. Specifically, for Condition 1, half of the infants saw female faces versus non-human objects, and the other half saw male faces versus non-human objects.

### 2.4. Reaching Behavior Coding

Infants’ reaching behaviors were recorded on a video camera positioned above and to the side of the infants. Infants who completed a minimum of 6 trials were included in the final sample. Any missing data were replaced with the sample average reaching for that particular trial. After the task was completed, experimenters coded the duration of each individual reach inside of the box with a stopwatch. Experimenters started timing once the infants’ third knuckle passed through the red curtain and into the box. Experimenters stopped timing once the fingers were fully removed from the box. Infant behaviors such as grabbing, pulling, or swiping at the front of the red curtain were not counted as reaches and thus not included in the duration data.

### 2.5. Analyses

For each condition, infants’ reaching duration inside of the box was used as the dependent measure. There were two test trial types: a single face presentation, and a two-face presentation. During the expected-empty outcome (i.e., one-face trials), reaching duration was coded when the experimenter gave the infant 10 s to explore the box after the first (and only) found face was taken away (see Figure 2). For the two-face test trials, there were two separate reaching duration periods coded. The first was during the 10 s pause after the experimenter took away the first (of two) found face. This was called the unexpected-empty outcome. The second coded reaching duration period was the 10 s following the researcher’s presentation and removal of the missing second face. This trial type was known as the expected-empty-final outcome. The primary comparison of interest was infants’ reaching duration for expected-empty versus unexpected-empty trials. Greater reaching during unexpected-empty trials has been interpreted as evidence of individuation of the two stimuli presented [49,50,51,59]. We also examined whether time spent with males and females or the number of gender related words infants knew (e.g., mom, aunt, dad, man) were related to reaching behavior. Infants were reported to spend an average of 67 percent of their time with females and 38 percent of time with males (percentages did not have to equate to 100 to account for shared time with both genders). Infants were reported to comprehend an average of 5.97 words (*SD* = 3.09) out of a possible 13 words. These measures were found to be unrelated to infant reaching and are not discussed further.

## 3. Results

### 3.1. Condition 1: Human vs. Non-human

#### 3.1.1. 12-Month-Olds

**Figure 3:**
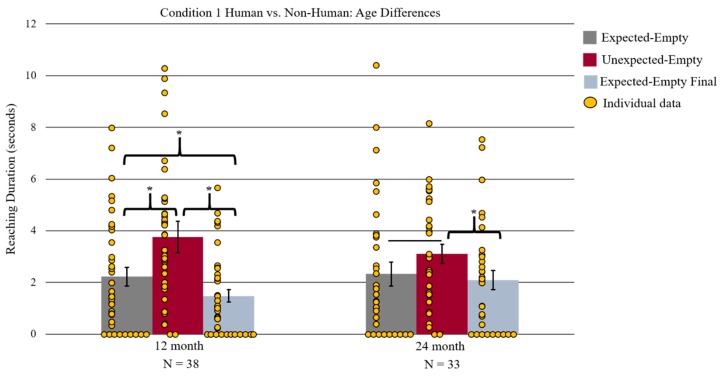
Depiction of the age separated two-way mixed measures ANOVA. Twelve-month-old infants reached significantly longer during the unexpected-empty relative to the expected-empty and expected-empty final outcomes. Additionally, infants showed longer reaching during the expected-empty than during the expected-empty final outcome. Twenty-four-month-old infants reached significantly longer during the unexpected- than during the expected-empty final outcome. Yellow circles represent each individual reaching data point. Error bars represent the standard error of the mean. The solid line represents a significant difference, which was not maintained following Bonferroni correction; * represents *p* < 0.05 Bonferroni corrected.

Infant’s sex did not significantly affect reaching duration and was not included as a variable in further analyses. A two-way mixed measures 3 × 2 ANOVA with face outcome (expected-empty, unexpected-empty, and expected-empty final) as a within-subjects variable and face stimuli race (Caucasian, African American) as a between-subject variable revealed a significant main effect of the face outcome *F*(2, 35) = 25.14, *p* < 0.001, ƞ^2^ = 0.59 (See Figure 3). The source of this main effect was examined by Bonferroni corrected paired-sample *t*-tests, which revealed significantly longer reaching during the unexpected-empty (*M* = 3.76 s, *SD* = 2.63) outcome relative to the expected-empty outcome (*M* = 2.22 s, *SD* = 2.20) and expected-empty final outcome (*M* = 1.48 s, *SD* = 1.58) *t*(37) = 3.76, *p* = 0.003, *t*(37) = 7.10, *p* < 0.001, respectively). Additionally, infants reached significantly longer during the expected-empty (*M* = 2.22 s, *SD* = 2.20) than the expected-empty final (*M* = 1.48 s, *SD* = 1.58) face outcome *t*(37) = 2.54, *p* = 0.045. The number of 12-month-old infants who reached longer during the unexpected than during the expected-empty outcome was significant (*n* = 30 out of 38; *p* < 0.001, two-tailed binomial test) as was the number of infants for unexpected- versus expected-empty final (*n* = 31 out of 38; *p* < 0.001, two-tailed binomial test). However, the number of infants who reached longer during the expected-empty than the expected-empty final outcome was not significant (*n* = 22 out of 38; *p* = 0.418, two-tailed binomial test). There were no other significant main effects or interactions found.

#### 3.1.2. 24-Month-Olds

Preliminary analyses found no effect of infant sex and was no longer included as a variable. The same two-way mixed measures 3 × 2 ANOVA used to analyze the 12-month-old data was conducted with the 24-month-old data. This ANOVA revealed a significant main effect of the face outcome *F*(2, 30) = 6.01, *p* = 0.006, ƞ^2^ = 0.29, which resulted from significantly longer reaching during the unexpected-empty (*M* = 3.10 s, *SD* = 2.10) compared to expected-empty final outcome (*M* = 2.09 s, *SD* = 2.11), as revealed by Bonferroni corrected paired-sample *t*-tests *t*(32) = 2.82, *p* = 0.024 (See Figure 3). Additionally, infants reached for a longer duration during the unexpected-empty versus the expected-empty outcome. However, this difference did not remain statistically significant following Bonferroni corrections *t*(32) = 2.27, *p* = 0.030, Bonferroni corrected *p* = 0.090. The number of infants who reached longer during the unexpected-empty versus expected-empty test was significant (*n* = 24 out of 33; *p* = 0.014, two-tailed binomial). However, the number of infants who reached longer during the unexpected-empty than the expected-empty final test only trended towards significance (*n* = 22 out of 33, *p* = 0.080, two-tailed binomial test).

**Figure 4:**
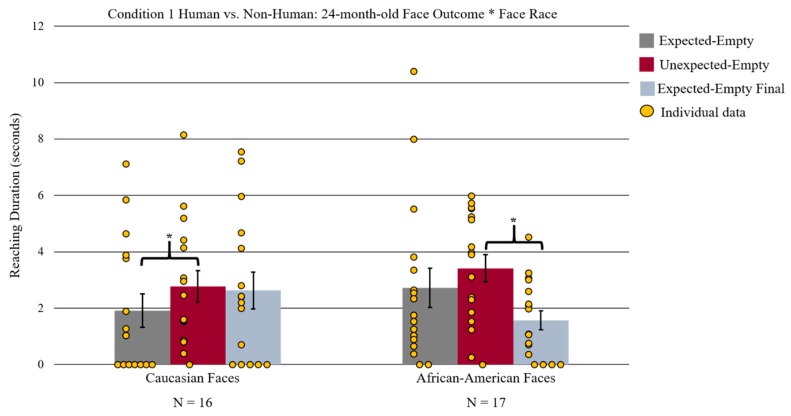
Depiction of the significant interaction of face outcome by face stimuli race found for the 24-month-old reaching duration. Infants reached significantly longer during unexpected-empty outcome than during the expected-empty outcome for Caucasian face stimuli but not for African American stimuli. Infants reached significantly longer during unexpected-empty versus expected-empty final outcome for African American face stimuli. Yellow circles represent each individual reaching data point. Error bars represent the standard error of the mean; * represents *p* < 0.05 Bonferroni corrected.

There was also a significant interaction of face outcome by the face stimuli, race *F*(2, 30) = 6.01, *p* = 0.050, ƞ^2^ = 0.18 (See Figure 4). This interaction was followed up with a paired-sample *t*-test (Bonferroni corrected) that compared the three levels of face stimuli outcomes separated by the face stimuli, race (Caucasian and African American). For Caucasian face stimuli, 24-month-old infants reached significantly longer during the unexpected-empty (*M* = 2.77 s, *SD* = 2.23) than during the expected-empty phase (*M* = 1.91 s, *SD* = 2.38) *t*(15) = 3.57, *p* = 0.009. Additionally, infants reached significantly longer for the expected-empty final (*M* = 2.63 s, *SD* = 2.61) than for the expected-empty outcome (*M* = 1.91 s, *SD* = 2.38) *t*(15) = −3.13, *p* = 0.021. In contrast, for African American face stimuli, infants only reached significantly longer during the unexpected-empty (*M* = 3.41 s, *SD* = 1.99) than during the expected-empty final (*M* = 1.57 s, *SD* = 1.39) outcome *t*(16) = 3.23, *p* = 0.015 but did not show significantly different reaching for the unexpected-empty relative to the expected-empty outcome *t*(16) = 1.10, *p* > 0.05. There were no other significant effects or interactions found.

To assess whether the differences in reaching duration patterns found between the two age groups were robust for direct comparison, we conducted a post-hoc three-way mixed measures 3 × 2 × 2 ANOVA with face outcome (unexpected-empty, expected-empty, and expected-empty final) as within-subjects variables. Infant age (12 months and 24 months) and face stimuli race (Caucasian and African American) were between-subject variables. This ANOVA revealed a significant main effect of face outcome *F*(2, 66) = 24.58, *p* < 0.001, ƞ^2^ = 0.43. Planned-comparison *t*-tests confirmed the source of this main effect. Infants reached significantly longer during the unexpected-empty (*M* = 3.45 s, *SD* = 2.41) than during the expected-empty (*M* = 2.27 s, *SD* = 2.40) and expected-empty final (*M* = 1.76 s, *SD* = 1.86) face outcomes *t*(70) = 4.35, *p* < 0.001, *t*(70) = 6.77, *p* < 0.001, which were Bonferroni corrected. The number of infants who demonstrated longer reaching during the unexpected-empty compared to expected-empty (*n* = 54 out of 71, *p* < 0.001, two-tailed binomial test) and expected-empty final (*n* = 53 out of 71, *p* < 0.001, two-tailed binomial test) face outcome was significant. The main effect was mediated by a significant two-way interaction of face outcome by infant age *F*(2, 66) = 3.74, *p* = 0.029. The same Bonferroni corrected planned-comparison *t*-tests that were previously reported for the age-separated analyses were conducted again as follow-up analyses for the two-way interaction. The exact same values and results were found, such that 12-month-old infants reached significantly longer during unexpected-empty than during the expected-empty and during the expected-empty final face outcome. In contrast, 24-month-old infants reached significantly longer during the unexpected-empty relative to the expected-empty final face outcome, but not for the critical comparison of unexpected-empty versus expected-empty face outcome. No other significant effects or interactions were found.

### 3.2. Condition 2: Female vs. Male

#### 3.2.1. 12-Month-Olds

**Figure 5:**
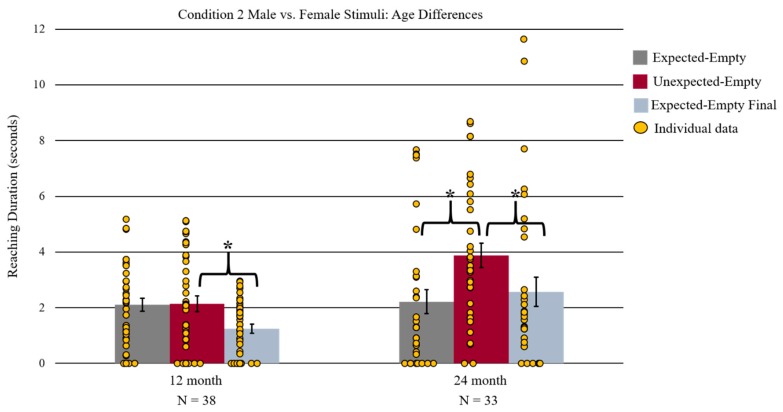
A depiction of age differences in reaching duration patterns for Condition 2. Twelve-month-old infants reached longer during unexpected-empty versus expected-empty final. In contrast, 24-month-old infants, but not 12-month-old infants, reached significantly longer during the unexpected-empty compared to expected-empty and expected-empty final. Error bars represent standard error of the mean, * represents *p* < 0.05 Bonferroni corrected.

Preliminary analyses revealed no effect of infant sex. Thus, we did not include it in further analyses. A 2-way 3 × 2 mixed measures ANOVA was conducted with face outcome (expected-empty, unexpected-empty, and expected-empty final) as a within-subject factors and the races of face stimuli (Caucasian, African American) were between-subject factors. The 2-way ANOVA revealed a significant main effect of face outcome *F*(2, 35) = 6.96, *p* = 0.003, ƞ^2^ = 0.29 (see Figure 5). Bonferroni corrected planned-comparison *t*-tests revealed the source of this main effect with significantly longer reaching duration during the unexpected-empty outcome (*M* = 2.14 s, *SD* = 1.74) compared to the expected-empty final outcome (*M* = 1.25 s, *SD* = 0.99) *t*(37) = 3.27, *p* = 0.006. Additionally, there was significantly longer reaching during the expected-empty (*M* = 2.11 s, *SD* = 1.42) versus expected-empty final (*M* = 1.25 s, *SD* = 1.42) *t*(37) = 3.46, *p* = 0.003. There was no statistically significant difference in reaching duration between unexpected-empty and expected-empty outcomes. The number of infants who reached significantly longer during unexpected-empty compared to expected-empty final was not significant (*n* = 24 out of 38, *p* > 0.05, 2-tailed binomial test), however the number of infants who reached longer during expected-empty versus expected-empty final was significant (*n* = 28 out of 38, *p* = 0.005, 2-tailed binomial test).

There was also a marginally significant 2-way interaction of face outcome and face stimuli race *F*(2, 35) = 3.24, *p* = 0.051, ƞ^2^ = 0.16. This interaction was followed-up with planned-comparison Bonferroni corrected *t*-tests separated by face stimuli age. For Caucasian faces 12-month-old infants displayed significantly longer reaching during unexpected-empty (*M* = 2.94 s, *SD* = 1.68) compared to expected-empty final (*M* = 1.50 s, *SD* = 1.01) *t*(19) = 3.40, *p* = 0.009. Additionally, infants reached significantly longer during expected-empty (*M* = 2.44 s, *SD* = 1.12) compared to expected-empty final (*M* = 1.50 s, *SD* = 1.01) *t*(19) = 2.74, *p* = 0.039. However, 12-month-old infants did not demonstrate statistically significant differences in reaching duration between the critical comparison of unexpected-empty (*M* = 2.94 s, *SD* = 1.68) and expected-empty (*M* = 2.44 s, *SD* = 1.12) *t*(19) = 1.80, *p* > 0.05. In contrast, infants who saw African American male and female faces did display any significant differences in reaching. There were no other significant effects or interactions found.

#### 3.2.2. 24-Month-Olds

No effects of infant sex were found and, thus, infant sex was not included as a variable in the primary analyses. The same two-way mixed measures 3 × 2 ANOVA used to analyze the 12-month-old data was again used for the 24-month-old data. There was a significant main effect of the face outcome *F*(2, 30) = 7.70, *p* = 0.002, ƞ^2^ = 0.34 (see Figure 5). Bonferroni corrected planned-comparison *t*-tests determined the source of this main effect with a significantly longer reaching duration during the unexpected-empty (*M* = 3.87 s, *SD* = 2.52) relative to the expected-empty (*M* = 2.21 s, *SD* = 2.46) and expected-empty final outcome (*M* = 2.57 s, *SD* = 3.02) *t*(32) = 3.86, *p* = 0.003; *t*(32) = 2.61, *p* = 0.042, respectively). The number of infants who displayed this longer reaching for unexpected-empty versus expected-empty and expected-empty final was significant (*n* = 27 out of 33, *p* < 0.001; *n* = 23 out of 33, *p* = 0.035, two-tailed binomial test respectively).

To assess whether the differences in reaching duration patterns found between the two age groups were robust for direct comparison, we conducted a post-hoc three-way mixed measures 3 × 2 × 2 ANOVA with face outcome (unexpected-empty, expected-empty, and expected-empty final outcome) as within-subjects variable and infant age (12 months and 24 months) and face stimuli race (Caucasian and African American) as between-subject variables. This ANOVA revealed a significant main effect of face outcome *F*(2, 66) = 9.58, *p* < 0.001, ƞ^2^ = 0.23, which resulted from a longer reaching duration for unexpected-empty (*M* = 2.94 s, *SD* = 2.30) relative to expected-empty (*M* = 2.16 s, *SD* = 1.96) and expected-empty final (*M* = 1.86 s, *SD* = 2.26) outcomes. This main effect was mediated by a significant two-way interaction between face outcome and infant age *F*(2, 66) = 7.10, *p* = 0.002, ƞ^2^ = 0.18. The same Bonferroni corrected planned-comparison *t*-tests that were previously reported for the age-separated analyses were conducted again as follow-up analyses for the two-way interaction. The exact same values and results were found, such that 12-month-old infants reached significantly longer during the unexpected-empty than during the expected-empty final outcome, but not for the critical comparisons of unexpected-empty versus expected-empty outcomes. In contrast, 24-month-old infants reached significantly longer during unexpected-empty relative to expected-empty and expected-empty final outcomes (see Figure 5).

**Figure 6:**
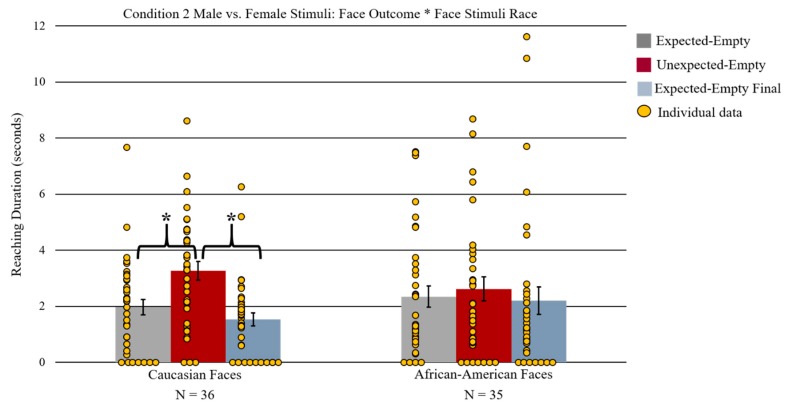
The graph depicts differences in reaching duration between face outcome types separated by face stimuli race. Infants who saw Caucasian faces reached significantly longer during the unexpected-empty than during the expected-empty and expected-empty final outcome. Infants who saw African American faces did not show significant differences in reaching duration. Error bars represent the standard error of the mean; * represents *p* < 0.05 Bonferroni corrected.

Lastly, there was a statistically significant two-way interaction of face outcome by face stimuli race *F*(2, 66) = 3.74, *p* = 0.029 (see Figure 6). Paired-sample *t*-tests separated by face stimuli race (Caucasian and African American) were conducted to follow up this interaction. Infants who saw Caucasian female versus male faces displayed significantly longer reaching during the unexpected-empty face outcome (*M* = 3.26 s, *SD* = 2.00) compared to the expected-empty (*M* = 1.98 s, *SD* = 1.64) and expected-empty final outcome (*M* = 1.53 s, *SD* = 1.42) *t*(35) = 4.23, *p* < 0.001 Bonferroni corrected, *t*(35) = 4.86, *p* < 0.001 Bonferroni corrected). There were no significant differences in reaching duration between expected-empty and expected-empty final outcomes. In contrast, when infants were presented with African American female versus male faces, follow-up planned-comparison *t*-tests revealed no significant differences in reaching between any of the three face outcomes.

## 4. Discussion

The present study examined whether infants possess kind-based representations for human versus non-human as well as male versus female faces using a manual search individuation task. Overall, findings indicate that 12- and 24-month-old infants represent human faces as distinct from non-human shapes across two different race face stimuli groups. Twenty-four-month-old infants also provided evidence that they individuated female versus male faces. The findings from the present study replicate and extend prior research on the development of kind-based representations of objects and people. This study was the first to use realistic human faces to test for kind-based individuation and to find that by their second birthday, infants can use face gender as a salient feature to track distinct individuals through time and space.

The current research was motivated by an interest in understanding factors that contribute to the development of social group awareness. The human face is one of the most salient social stimuli we perceive, recognize, and categorize starting from a very young age [63]. Furthermore, processing social stimuli, such as faces, has critical consequences in learning individual identities, social cues, language, and other socio-emotional capabilities. However, there is limited work examining if and when infants develop kind categories for different types of face groups. The data presented in this current study begins to address this limitation in the literature by presenting evidence for conceptual representations for both superordinate and subordinate groups of human faces in a sample age older than perceptual narrowing during infancy, yet prior to the social biases observed during preschool years.

One interpretation of the current data is that infants successfully individuated the faces by representing the underlying kind category they belonged to (e.g., ‘human’ and ‘male’ or ‘female’). However, an alternative interpretation of the present findings is that infants were individuating the different identities of faces that were used in our stimuli set. After all, the face perception literature shows that even newborn infants can perceive differences between two face identities [6,64], making it possible that infants in the present study did the same. If this were the case, we would have expected infants in both age groups to have individuated all of the different types of faces we presented. If infants simply used face identity, then it would have been expected that younger infants would individuate male and female faces just as older infants did. The face stimuli we employed [62] had been validated as clear representations of the gender and race groups that each individual self-identified as. Furthermore, it is not possible to clearly argue that the differences in face identities were more distinct to infants than the differences represented across the kind categories of human and gender. Thus, we argue that it is more likely that the current data supports infants individuating faces using higher-order kind representations that are not strictly perceptual or based solely on differences in face identity.

### 4.1. Infants’ Representation of Human-Face Kinds

The current study replicated and extended previous findings that infants possess the superordinate kind-category ‘human’ and can individuate human faces as distinct from non-human faces. Previously, 10-month-old infants succeed in tracking a human doll’s face as a distinct individual when paired with an animal face [57]^,^ as well as an inverted face [58]. Based on these findings, the “human first” hypothesis proposes that human face individuation is a primitive distinction acquired before infants possess finer-grained categories. The present study extends past research findings in two meaningful ways. First, methodologically, the present study used realistic human faces that were more ecologically valid than those previously used. In the present study we also varied the number of face identity examples and the types of human faces tested against non-human faces. This allowed for greater generalization of our findings. Second, we tested whether a human face would be represented as an ontological kind across different race groups. Previous perceptual research suggests that within the first year of life infants narrow their processing expertise to faces that represent the race they have the most experience with [4]. Additionally, 9-month-old infants will form a specific own-race face category but will create a general other-race face category that includes any face of a race that is unfamiliar to them [30]. Prior to the current study, it was unclear if infants’ formation of a general other-race face category also meant that faces from a racial out-group were not conceptually represented as human faces. We found that 12-month-old infants equally individuated a human face from a non-human face for both Caucasian and African American faces. Interestingly, evidence from the 24-month-old infants indicates that infants individuated Caucasian faces but may not have been doing so for African American faces. This was an unexpected result and one that needs further examination. It is possible that this interaction of race reflects some type of saliency bias for these older infants that could be related to developmental changes observed for categorizing other-race faces [30]. It is important to note that the majority of our infants in this sample were Caucasian and spent most of their time with same-race individuals. Future work should examine whether race-based experiences or group membership influence older infants’ kind-based individuation of faces varying by race. Overall our findings support the conclusion that infants use human faces as salient markers for representing and tracking individuals within a scene and that these features connect faces in a meaningful way beyond observable variation in surface features, such as race.

### 4.2. Infants’ Kind-Based Individuation and Gender

Another aim of the current study was to assess infants’ individuation of human faces based on finer-grained features that reflect social group membership. Research from infant face processing, as well as the social cognitive development in children, provided the current project with the grounds to assess individuation based on a kind concept of gender. Early in infancy, there is a strong visual preference for faces that reflect the gender of one’s primary caregiver [5]. Gender is hypothesized to be a more salient marker of group membership and is categorized by children differently from race [33,61,65,66,67,68,69]. Interestingly, toddlers between 19 and 26 months of age recognized the category of gender but only with the use of novel auditory labels [70]. The authors proposed that the formation of categories without the use of labels may be a more fragile capability for infants at this age. Studies have shown that between 3- and 5-year-old children develop differential processing and essentialist thinking for gender [71,72]. For example, 5-year-olds make judgments indicating their beliefs that gender, but not race or ethnicity, are biologically inherited [71,73]. Additionally, 5- to 7-year-old children reason that gender, but not race, is a ‘natural kind’ that is used to conceptually group individuals together and use this kind category to predict behavior [68,74]. Combined with the present findings, it appears that gender is a social category that is quickly encoded and begins to develop into childhood.

The critical difference between individual faces presented in a two-face outcome in Condition 2 was the gendered facial features, with one face having a stereotypical male appearance and the other a stereotypical female appearance. Experimenters did not provide any information to the infants about specific features they should attend to or label each face differently during the task. The current findings indicate that the perceptual or observable features that distinguish male and female faces were not thought of as interchangeable during the course of the task. Infants’ individuation of these faces suggests that gendered features are not only perceptually salient but have a deeper, more meaningful distinction that separates one face from another. Similar to human versus non-human face individuation across two race groups, we present evidence for individuation of face gender of Caucasian and African American faces. However, our results also indicate that infants overall were more robust at individuating Caucasian relative to African American faces. These findings are consistent with previous face perception studies that show perceptual biases for female faces of a highly familiar race relative to unfamiliar race groups [20,21,23,24]. Our participant sample was primarily Caucasian, which may be a related factor for why we found some differences in reaching performance across the two face race stimuli groups. Future studies will be needed to further examine whether an infants’ racial or ethnic background and/or experiences with members of other races influence their development of individuation of faces. Evidence for the kind-category of gender has not been previously seen prior to early childhood and through the use of more complex decision-making tasks with preschoolers [68,74]. To our knowledge, this is the first evidence that indicates even 2-year-old infants have a kind-category for gender.

To some extent, our findings that 24- but not 12-month-olds individuate females and males seems surprising compared to the conclusions drawn from the face perception literature. With such a strong female face bias seen as early as 3 months of age [5,6], why might this not translate into younger infants also using gender cues to individuate faces? One important consideration is that perceptual studies typically present faces simultaneously and do not require infants to hold one distinct individual in their working memory while also tracking a second individual. Thus, it is possible that although younger infants can separate a human from a non-human, male and female facial recognition is not yet salient enough for infants to separate into distinct individuals. It is also unclear when, specifically, infants between ages one and two start to readily individuate female and male faces. Future work is needed to further examine the developmental shift that occurs from 1 to 2 years of age and the presence of using gendered facial features to individuate people.

### 4.3. Connecting Perception to Conceptual Representations

By investigating conceptual representations of face gender, the present study tries to take initial steps in bridging an information gap between infants’ perceptual processes of faces and later occurring socio-cognitive understanding of social groups. The present data offers evidence that gender is a kind-based marker of distinct groups. It is plausible that infants’ acquisition of the concept for gender groups is a continuous process that first starts to transition from perception to conceptual representation around and infant’s second birthday. The present work may also contribute to our understanding of developmental origins for social group biases. Social judgments for gender are present in early childhood. These biases may in part develop from infants’ early perceptual biases for different types of faces during infancy. However, there is limited research that provides data to fully describe potential precursors or developmental trajectory of social group biases that are observed by 3 or 4 years of age. One hypothesis is that biases, particularly implicit biases, begin at the onset of having the concept of group membership [66,75]. Although we cannot offer evidence that infants in our sample have awareness of their own membership to a gender group, it seems that by about 2 years of age, infants use representations of maleness and femaleness to separate faces. This, in turn, could be the start of an infant’s concept of group membership and the onset of implicit group biases. The present data indicate that a socio-cognitive process of individuating people into different categories occurs even before the experience of formal schooling and may mark an important period when infants are sensitive to information that denotes membership in different social categories.

### 4.4. Future Directions

This study is the first to examine infants’ kind-based individuation for faces based on gender. There are several opportunities to expand on the present findings. Additional measures of infants’ visual attention towards faces during the box task would benefit our understanding of the way time spent looking at a face may underlie infants’ processing and encoding of the faces they individuate. Due to the current methodology, it is not clear if infants significantly varied in the total duration of visually attending to the presented stimuli. The experimenters made explicit attempts to direct infants’ visual attention towards the faces while they were presented (as described in the procedure section). Past research indicates that as early as 3 months of age, infants show a biased looking time toward female faces [5]. It is possible in the present study that there were differences in the strategies or time spent looking at the presented faces. This type of visual attention will need to be further examined through the use of technology, such as head-mounted eye-tracking systems that allow infants to engage with their environments while tracking where they are looking.

For gender kind representations, it is unclear which feature or multiple features of the faces were used to conceptually distinguish between male and female faces. From face perception literature, there is evidence that hairline is a critical feature for young infants to perceptually detect differences between male and female faces [19]. In the present study, most of the female faces used in the gender individuation condition had longer hair than the male faces. Thus, this could be a critical cue for infants to individuate males from female faces and be part of their understand of ‘boy’ and ‘girl.’ Another open question is whether infants’ individuation of human versus non-human faces and/or males and females are early markers for infants beginning to have self-representations in relation to social group membership. Might infants first need to use facial features to recognize meaningful connections between individuals as a way to build an understanding of one’s own group membership? Lastly, the current study found that infants develop a sensitivity to the distinction between male and female faces by the age of two. One open question is whether this pattern would be found in households where infants are raised by gender non-binary caregivers. As infants and children have increased exposure to gender non-binary individuals, might the male–female conceptual dichotomy change? A potential follow-up experiment could assess the flexibility of older infants’ representations of the gender binary by examining whether infants with gender non-conforming caregivers show a similar or possibly weaker pattern of individuation for female versus male faces. Each of these open questions are in need of future research to further support our understanding of how infants think about, and may treat, people of different gender groups.

## 5. Conclusions

The present study has taken initial steps in further investigating infants’ individuation of faces based on the socially salient marker of face gender. The present data provide evidence that by 12 months of age, infants possess the capability to individuate faces based upon the kind ‘human’ they see. By 2 years of age, infants also demonstrated the capability to use gender to track the number of distinct individuals involved in a presented scene. By investigating whether infants have kind concepts for gender, we aimed to bridge the gap in our understanding of when the concepts of in-group and out-group membership begin to develop. Our goal is that this type of work will only be the beginning of a line of research that will better understand the connection between perceptual biases and later social prejudices.
